# The relationship between college students’ autonomous fitness behavior and mental health literacy: chain mediating effect test

**DOI:** 10.3389/fpsyg.2025.1665652

**Published:** 2025-10-09

**Authors:** Chunping Chen, Bin Wang

**Affiliations:** ^1^School of Physical Education, China University of Mining and Technology, Xuzhou, China; ^2^Department of Physical Education and Sports, Beijing Union University, Beijing, China

**Keywords:** autonomous fitness behavior, mental health literacy, self-control, exercise identity, college students

## Abstract

**Objective:**

This study aimed to explore the effect of autonomous fitness behavior on college students’ mental health literacy, and the mediating roles of self-control and exercise identity.

**Methodology:**

A cross-sectional survey using cluster sampling was conducted among 974 college students from Shandong Province, China. Data on autonomous fitness behavior, mental health literacy, self-control, and exercise identity were collected using standardized scales (Cronbach’s *α*: 0.722–0.949). SPSS 26.0 (PROCESS Macro Model 6) and AMOS 26.0 were used for statistical analyses, including correlation, regression, and mediation effect tests with 5,000 Bootstrap samples.

**Results:**

(1) Autonomous fitness behavior positively predicted mental health literacy (*β* = 0.416, *p* < 0.001), self-control (*β* = 0.301, *p* < 0.001), and exercise identity (*β* = 0.198, *p* < 0.001); self-control also positively predicted exercise identity (*β* = 0.281, *p* < 0.001). (2) Three significant indirect paths were identified: ① Autonomous fitness behavior → Self-control → Mental health literacy (indirect effect = 0.024, 95% CI [0.008, 0.042], accounting for 5.77% of total effect); ② Autonomous fitness behavior → Exercise identity → Mental health literacy (indirect effect = 0.155, 95% CI [0.122, 0.189], accounting for 37.25% of total effect); ③ Autonomous fitness behavior → Self-control → Exercise identity → Mental health literacy (indirect effect = 0.066, 95%CI[0.049, 0.084], accounting for 15.87% of total effect). The total indirect effect was 0.245 (95% CI [0.210, 0.279]).

**Conclusion:**

Among the surveyed college students, autonomous fitness behavior influences mental health literacy directly and indirectly through the independent mediating effects of self-control and exercise identity, as well as their chain mediating effect. These findings provide preliminary evidence for the mechanism linking autonomous fitness behavior to mental health literacy, which may inform targeted mental health interventions in higher education settings.

## Introduction

In recent years, mental health challenges among Chinese university students have garnered increasing attention within educational and public health sectors. Academic pressures, social adaptation difficulties, and pervasive digital lifestyles contribute significantly to psychological distress, particularly among lower-grade students who often lack adequate mental health literacy-a core competency for recognizing, managing, and promoting psychological well-being (Su et al., 2024).

Autonomous fitness behavior, characterized by self-determined and intrinsically motivated physical activity (Na et al., 2024), has shown promise in enhancing mental health outcomes. However, existing research has predominantly examined general physical activity, overlooking the specific role of autonomy and the psychological mechanisms through which autonomous fitness behavior may improve mental health literacy. This gap is especially salient in the context of Chinese higher education, where cultural and institutional factors may shape these relationships uniquely.

Self-control and exercise identity represent two potential mediators in this relationship. Self-control enables individuals to maintain consistent exercise habits by regulating impulses and supporting goal-directed behavior (Zhang S. et al., 2025; Zhang L. C. et al., 2025), while exercise identity reflects the integration of an “exerciser” role into one’s self-concept, enhancing intrinsic motivation and psychological resources related to mental health (Dong et al., 2024). Moreover, these factors may operate in sequence: autonomous fitness behavior could bolster self-control, which in turn facilitates the development of exercise identity, ultimately contributing to autonomous fitness behavior.

### The relationship between autonomous fitness behavior and mental health literacy

Mental health literacy-defined as the knowledge, beliefs, and competencies needed to identify, address, and promote psychological well-being (Jorm, 2012) is critical for enhancing psychological resilience in university students. Recent years have seen heightened mental health concerns in this population due to academic pressures, social integration challenges, and career uncertainties, with over 30% of Chinese university students showing clinically significant anxiety or depression symptoms (Cheng, 2021). This highlights the need to identify effective protective factors.

Physical activity, particularly autonomous fitness behavior, has emerged as a promising candidate. Autonomous fitness behavior refers to self-directed, voluntary exercise driven by intrinsic motivation (Jacobs and Jacobs, 2000), differing from externally regulated activities (e.g., compulsory physical education). Its proactive, choice-based nature may strengthen positive mental health effects by fostering autonomy and commitment. Existing research supports a positive link between physical activity and mental health literacy: consistent exercise improves emotional regulation, self-efficacy, and stress coping-key dimensions of mental health literacy (Biddle et al., 2018). For university students, regular fitness engagement correlates with higher self-esteem and lower psychological distress (Zhang S. et al., 2025). However, a critical gap remains: few studies have examined how the autonomous nature of exercise moderates these effects, despite evidence that motivation quality influences physical activity’s psychological outcomes.

Self-Determination Theory (SDT) provides a robust framework to address this gap. SDT distinguishes between autonomous motivation (driven by personal interest or valued goals) and controlled motivation (driven by external pressure), positing that autonomous engagement better promotes psychological well-being (Ryan and Deci, 2000). Applied to physical activity, SDT suggests autonomously motivated exercise enhances feelings of competence and self-determination, strengthening individuals’ ability to manage mental health challenges. Empirically, Chinese university students with voluntary fitness engagement score higher on mental health literacy assessments-especially in stress management and self-acceptance-han those with externally motivated exercise (Zhang L. C. et al., 2025).

Additionally, autonomous fitness behavior supports long-term physical activity adherence, which is key to sustained mental health improvements. Maintaining self-regulated exercise routines boosts psychological flexibility (Öztekin et al., 2025) and acts as a protective factor against academic stress and social isolation in university students, Longitudinal studies further show that students with autonomous fitness behavior exhibit steady, progressive improvements in mental health literacy compared to peers with less self-determined exercise patterns (Min et al., 2018).

To address current limitations, this study examines the direct relationship between autonomous fitness behavior and mental health literacy among Chinese college students. Clarifying this association will provide empirical evidence to inform targeted interventions that use autonomous fitness to enhance college students’ mental health literacy. Based on the above, Hypothesis 1 is proposed: Autonomous fitness behavior can positively predict college students’ mental health literacy.

### The mediating role of self-control

Self-control, defined as the capacity to regulate one’s impulses, emotions, and behaviors in pursuit of long-term goals (Duckworth and Gross, 2014), has become a critical construct for understanding human behavior-particularly within health-related activities. Within the association between autonomous fitness behavior and mental health literacy, self-control may function as a significant mediator.

Research has highlighted close links between self-control, physical activity, and mental health. For example, a study involving Chinese college students found that higher self-control levels were associated with greater adherence to autonomous fitness plans (Na et al., 2024). Individuals with stronger self-control were more adept at overcoming barriers such as fatigue or low motivation, thereby maintaining regular engagement in fitness activities. This consistent participation in autonomous fitness, in turn, contributed to improved mental health literacy-including enhanced skills in stress management and emotional regulation.

In the domain of mental health, self-control has also been linked to better psychological well-being. A cross-sectional study focusing on adolescents indicated that self-control correlated positively with psychological well-being and negatively with mental health problems (Gkalp, 2023). Notably, resilience-a key component of mental health literacy-was found to mediate the relationship between self-control and mental health, suggesting that self-control may influence mental health outcomes by strengthening individual resilience.

Nevertheless, the complex interactions among autonomous fitness behavior, self-control, and mental health literacy remain underexplored, especially among Chinese college students. Clarifying how self-control mediates the relationship between autonomous fitness and mental health literacy could yield valuable insights for developing effective interventions. Such interventions might target self-control enhancement as a means to promote both autonomous fitness behavior and mental health literacy in this population. Based on the above research, Hypothesis 2 is proposed:autonomous fitness behavior affects college students’ mental health literacy by improving self-control.

### The mediating role of exercise identity

Exercise identity is defined as the extent to which an individual perceives themselves as an exerciser, integrating fitness behavior into their self-concept and value system (Strachan et al., 2025). This psychological construct encompasses emotional attachment, behavioral commitment, and social recognition related to physical activity, acting as a stable internal driver for sustained exercise participation.

Recent studies have underscored the close association between autonomous fitness behavior and exercise identity. When college students engage in fitness activities out of intrinsic motivation-such as personal interest or growth-they are more likely to develop a strong exercise identity (Bennett and Mekler, 2024). Research has found that autonomous fitness participation significantly predicts the formation of exercise identity, with individuals increasingly describing themselves as “people who exercise regularly” (Auckland, 2024). This suggests that voluntary, self-directed fitness behavior fosters a deeper psychological connection with physical activity, thereby strengthening one’s identity as an exerciser.

Exercise identity, in turn, plays a pivotal role in enhancing mental health literacy. Studies indicate that a strong exercise identity is associated with higher self-efficacy, improved emotional regulation, and better social adaptation-all key components of mental health literacy (Cui and Zhou, 2025). Specifically, individuals who strongly identify as exercisers tend to develop a sense of mastery: consistent fitness engagement provides tangible evidence of their ability to set and achieve goals, which boosts their confidence in managing psychological challenges (Su et al., 2024). For college students, this may translate to more effective coping strategies when facing academic stress or interpersonal conflicts.

The mediating role of exercise identity thus lies in its capacity to transform autonomous fitness behavior into a stable self-concept, which in turn nurtures mental health literacy. Autonomous fitness lays the groundwork for the formation of exercise identity, and this identity reinforces the psychological resources essential for maintaining mental well-being. Exploring this pathway among Chinese college students can provide insights into how fitness programs can be leveraged to promote not only physical health but also psychological resilience. Based on the above research, Hypothesis 3 is proposed: autonomous fitness behavior affects college students’ mental health literacy by improving exercise identity.

### The chain mediating effect of self-control and exercise identity

Beyond their independent mediating roles, self-control and exercise identity may form a sequential pathway through which autonomous fitness behavior influences college students’ mental health literacy. This chain mediating effect suggests that autonomous fitness behavior first enhances self-control, which in turn strengthens exercise identity, ultimately contributing to improved mental health literacy.

Self-control serves as a foundational psychological resource for the development of exercise identity. The mechanism through which self-control underpins exercise identity is fundamentally a dynamic process involving the accumulation of identity-verification cues via sustained self-regulatory behavior. Specifically, when college students leverage self-control to overcome immediate temptations-such as resisting sedentary forms of entertainment or prioritizing time for physical activity-to maintain consistent fitness engagement, they acquire two pivotal types of experiences. First, they develop a sense of behavioral consistency: regular fitness participation transforms physical activity from an “occasional choice” into a “daily habit,” and this behavioral stability provides tangible evidence for embedding the “exerciser” role within their self-concept. Second, they gain a sense of competence: throughout sustained fitness involvement, individuals may perceive improvements in physical fitness or achieve predefined fitness goals. These concrete outcomes reinforce the cognitive belief that “I possess the ability to persist in exercise,” which in turn translates into emotional identification with the “exerciser” identity. This process aligns with the core logic of “behavioral verification–identity reinforcement” in identity construction theory (Stets and Burke, 2014a,b). Repeated role-consistent behaviors-specifically, fitness behaviors sustained through self-control-continuously validate an individual’s preexisting cognitive schema of the “exerciser” identity. Over time, this iterative verification ultimately internalizes the “exerciser” identity, shifting it from a mere “external label” to a deeply rooted “core self-trait.”

Individuals with higher self-control are better able to maintain consistent fitness participation, even when faced with obstacles such as time constraints or fatigue (Englert, 2025). This sustained engagement provides repeated opportunities to reinforce the connection between fitness behavior and self-concept, gradually solidifying exercise identity. Student who successfully resists the temptation to skip workouts through self-control may increasingly view themselves as “someone who exercises regularly” thereby strengthening their exercise identity. A strengthened exercise identity, in turn, acts as a catalyst for improving mental health literacy. When fitness behavior becomes a core part of one’s self-identity, individuals are more likely to derive psychological benefits-such as enhanced self-efficacy and emotional stability-from their exercise routines (Slyke et al., 2021). These benefits, rooted in a strong sense of exercise identity, further boost their capacity to understand, manage, and promote mental well-being.

The chain mediating effect of self-control and exercise identity thus creates a virtuous cycle: autonomous fitness behavior fosters self-control, which strengthens exercise identity, and this enhanced identity ultimately elevates mental health literacy. This sequential pathway underscores the complex psychological mechanisms through which physical activity influences mental well-being, highlighting that the combined effect of self-control and exercise identity exceeds their individual contributions. Based on the above research, Hypothesis 4 is proposed: Self-control and exercise identity have a chain mediating effect between autonomous fitness behavior and college students’ mental health literacy.

**Figure 1:**
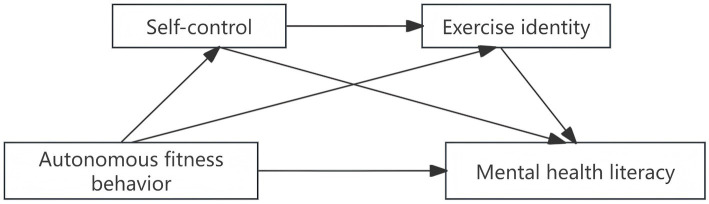
Conceptual model.

Accordingly, the present study aims to construct a chain mediation model (Figure 1) to examine the impact of Chinese college students’ autonomous fitness behavior on their mental health literacy, while clarifying the chain mediating role of self-control and exercise identity in this relationship. The scientific value of this work lies in its focus on autonomy-driven mechanisms and sequential psychological processes, offering an empirical basis for designing targeted student mental health interventions.

Through testing these hypotheses, we seek to provide theoretical and practical insights into how autonomous physical activity can be leveraged to enhance mental health literacy in Chinese university settings.

## Materials and methods

### Procedure and participants

This study employed a cluster sampling approach, systematically selecting one public school from each of the eight regions across the eastern, central, and western areas of Shandong Province, China. From each selected school, three classes were randomly sampled from the freshman cohort, with each class consisting of 50 students. In total, 1,000 questionnaires were distributed. All participants belonged to the same ethnic group and had similar socioeconomic statuses. In this study, students with severe physical or mental illnesses (e.g., depression diagnosed by a hospital), those unable to participate in fitness activities, and those with incomplete questionnaire responses were excluded. Notably, the gender composition of the sample (691 females, 70.9%; 283 males, 29.1%) is consistent with the overall gender distribution of freshmen in the selected colleges and universities-this is attributed to the cluster sampling method that randomly selected intact classes, avoiding intentional overrepresentation of either gender and thus minimizing sampling bias related to gender. After excluding invalid questionnaires due to response patterns, missing data, and other issues, 974 valid questionnaires were retained, resulting in a recovery rate of 97.4%. Among the valid responses, 691 participants were female (70.9%), and 283 were male (29.1%), with an average age of 19.4 ± 1.33 years. A differential test indicated no significant statistical differences among schools, thus justifying the analysis of the entire dataset. Sample size was determined using G*Power 3.1. For multiple regression analysis (four core variables), assuming an effect size of 0.15 (medium effect), *α* = 0.05, power (1-*β*) = 0.90, a minimum of 368 participants was required. To account for a potential 20% invalid questionnaire rate, 1,000 questionnaires were distributed. After excluding invalid ones, 974 valid responses were retained-meeting the pre-calculated statistical power requirement (Faul et al., 2009).

The study procedures were as follows: Prior to data collection, informed consent was obtained from school administrators, homeroom teachers, and individual participants, and researchers received unified training (on questionnaire interpretation and ethical norms) to ensure consistent guidance. Before questionnaire distribution, researchers further explained the study purpose, procedures, and confidentiality commitments to participants, and obtained written informed consent from each. Questionnaires-incorporating control variables (age, gender)-were group-administered on-site in quiet classrooms, with real-time question-answering by researchers and immediate collection post-completion to avoid missing data; data were collected from September 3 to November 1, 2024, with 30 min given per participant. For data quality control, questionnaires with >10% missing items, uniform responses, or obvious bias were excluded as invalid; valid data were double-entered by two independent researchers and cross-checked for accuracy.

### Measures and instruments

#### College students’ autonomous fitness behavior

To measure college students’ autonomous fitness behavior, this study employed the Self-determination Scale for Adolescents’ Autonomous Fitness Behavior developed by Rui (2013). The scale consists of three dimensions: self-determination (*α* = 0.859), self-support (α = 0.786), and self-regulation strategies (*α* = 0.876), comprising 43 items in total. The topics include such as: fitness is my hobby and I enjoy working out, I have good fitness knowledge and skills, During fitness activities, I always have plenty of energy and physical strength. A 5-point Likert scale was used, where 1 indicated “strongly disagree” and 5 indicated “strongly agree.” This scale has been applied in many domestic studies on adolescents and has good reliability and validity (Wang et al., 2025). In this research, the questionnaire demonstrated a high internal consistency reliability, with a Cronbach’s α coefficient of 0.759.

#### Positive mental health literacy(PMHL)

The Positive Mental Health Literacy Scale (PMHL) was developed by Bjrnsen et al. (2017). Chinese scholars Cui et al. (2024) revised it into a Chinese version. The scale is used to measure psychological literacy levels among college students. The scale consists of 10 items, including examples such as: Deal with stress in a good way, Believe in oneself, Develop good sleep habits. It uses a 5-point rating scale, where “completely disagree” is scored as 1 and “completely agree” as 5. Higher total scores indicate a higher level of positive mental health literacy among participants. The scale demonstrates strong reliability with a Cronbach’s *α* coefficient of 0.833 and excellent fit to the data, as evidenced by the following indices: χ^2^ = 67.702, df = 35, χ^2^/df = 1.934, GFI = 0.970, SRMR = 0.035, RMSEA = 0.047, and CFI = 0.969. In this research, the questionnaire demonstrated a high internal consistency reliability, with a Cronbach’s α coefficient of 0.949.

#### Multidimensional self-control scale

The Multidimensional Self-Control Scale (MSCS), developed by Nilsen et al. (2020), is designed to measure students’self-control abilities. The scale consists of 29 items, such as: I have let things drag on so long that it has affected my health or efficiency, It’s hard for me to start working, I have trouble concentrating. It uses a 5-point Likert scale, ranging from 1 (strongly disagree) to 5 (strongly agree), where higher scores indicate higher levels of self-control. Based on the original scale, the authors proposed a shortened version- MSCS-BMSCS. This abbreviated scale includes 8 items across two dimensions: inhibitory self-control and initiatory self-control. Dong et al. (2024) validated the Chinese version of the shortened scale. Results showed test–retest reliability of 0.795 for the total scale, with test–retest reliabilities of 0.792 and 0.649 for the subdimensions, respectively. Model fit indices were as follows: χ^2^/df = 3.973, GFI = 0.969, CFI = 0.940, AGFI = 0.906, and RMSEA = 0.076. In this study, the Cronbach’s *α* coefficient for the scale was 0.722, indicating high reliability.

#### Exercise identity scale(EIS)

The Exercise Identity Scale (EIS), originally developed by Anderson and Cychosz (1994), was revised by Menglong et al. (2019) to measure college students’ exercise identity. The scale comprises nine items, such as: I think I’m an exerciser. When I introduce myself to others, I mention that I exercise. I have a lot of exercise goals. The scale demonstrates test–retest reliability of 0.79 and a Cronbach’s *α* coefficient of 0.902. Model fit indices are as follows: χ^2^/df = 2.197, GFI = 0.909, CFI = 0.943, AGFI = 0.906, and RMSEA = 0.075. In this study, the Cronbach’s α coefficient was 0.938, indicating high internal consistency.

### Research procedure and statistical analysis

1. IBM SPSS 26.0 statistical software was used for data analysis, including descriptive statistics and correlation analysis of and other variables; Data were tested by common method bias.
2. Model 6 in the macro program PROCESS of SPSS was used to conduct the mediating effect test (Hayes, 2013). Major test: the direct effect autonomous fitness behavior and positive mental health literacy; Mediating effect of self-control and exercise identity; the chain mediating effect of autonomous fitness behavior and positive mental health literacy.
3. AMOS26.0 was used to test the fitting degree of the mediating model between autonomous fitness behavior and positive mental health literacy.

## Results

### Common method deviation test

This study was conducted using a questionnaire survey. To detect and control potential common method biases, we included 9 reverse items in the “Autonomous Fitness Behavior Scale” during the design of the questionnaire. During data collection, we ensured data accuracy through various methods such as on-site filling out, on-site answering questions, and on-site questionnaire collection. Additionally, to further verify the issue of common method biases, this study employed the Harman’s single-factor test method for data analysis. By extracting all items into a single-factor unrotated exploratory factor analysis, we found that there were 8 factors with eigenvalues greater than 1, with the largest factor explaining 38.31% of the variance. This proportion is lower than the 40% criterion proposed by Hair et al. (1998), indicating that there is no significant common method bias in this study. To control for bias, cluster sampling was used to cover 8 regions of Shandong Province, with the sample gender ratio matching the actual freshman gender distribution of selected universities to avoid regional or gender over representation (selection bias). For information bias, standardized training for researchers and on-site guidance minimized deviations in questionnaire interpretation; 9 reverse-scored items were included in the Autonomous Fitness Behavior Scale to reduce response bias; and double data entry with cross-checking ensured data accuracy. Regarding confounding bias, age and gender-variables that showed significant correlations with key variables in pre-analysis-were included as control variables in regression models to mitigate their potential confounding effects.

### Descriptive statistical and correlation analysis

Table 1 adopted Pearson correlation analysis to examine bivariate relationships among variables, primarily for two reasons: first, to preliminarily verify the directional associations between autonomous fitness behavior, mental health literacy, self-control, and exercise identity (consistent with the study’s preliminary hypothesis-testing purpose); second, to screen for potential confounding variables (e.g., age, gender) by assessing their correlations with key variables, laying a foundation for subsequent regression and mediation analyses. As shown in Table 1, the correlation coefficients of autonomous fitness behavior, mental health literacy, self-control and exercise identity are all statistically significant. The correlation analysis shows that autonomous fitness behavior is positively correlated with self-control, exercise identity and mental health literacy (*p* < 0.01). Also, there are Sex differences in age, autonomous fitness behavior, mental health literacy, self-control and exercise identity (*p* < 0.01), age has no correlation with each index.

**Table 1:** Descriptive statistics and correlation analysis.

Variables	*M*	SD	1	2	3	4	5	6
Gender	1.29	0.454	1					
Age	19.42	1.245	0.103					
Autonomous Fitness Behavior	3.528	0.741	0.039**	−0.066	1			
Mental health literacy	2.951	0.574	0.177**	−0.077	0.594**	1		
Self-control	3.695	0.632	0.093**	−0.068	0.506**	0.512**	1	
Exercise identity	4.463	1.065	0.197**	−0.061	0.520**	0.781**	0.515**	1

*N* = 974. *p < 0.05, **p < 0.01, ***p < 0.001.

Table 2 presents the correlation degrees of autonomous fitness behavior, mental health literacy, self-control, and exercise identity across different genders. Significant gender differences exist, specifically as follows: females exhibit higher levels of mental health literacy than males. Regarding autonomous fitness behavior, self-control, and exercise identity, males demonstrate significantly higher scores than females in all three dimensions.

**Table 2:** Differences in gender.

Variables	Gender	M ± SD	*t*	*p*
Autonomous fitness behavior	Female	3.44 ± 0.66	1.46	***
	Male	3.73 ± 0.88		
Mental health literacy	Female	2.88 ± 0.55	−7.66	**
	Male	3.13 ± 0.60		
Self-control	Female	3.66 ± 0.58	−3.52	***
	Male	3.79 ± 0.72		
Exercise identity	Female	4.49 ± 0.97	−6.63	***
	Male	4.39 ± 1.26		

*N* = 974. **p < 0.01, ***p < 0.001.

### Significance test of mediation effect

In this study, the model 6 of the SPSS plug-in PROCESS compiled by Hayes (2013) was used to test the mediation effect, with 5,000 Bootstrap samples employed for the testing. The results of regression analysis are shown in Table 3, with mental health literacy as the dependent variable, self-control and exercise identity as the intermediary variables, and age, gender as the control variables.

**Table 3:** Regression analysis of the relationship between variables.

Variables	Model 1 Mental health literacy			Model 2 Self-control			Model 3 Exercise identity			Model 4 Mental health literacy		
	*β*	*t*	95% CI	*β*	*t*	95% CI	*β*	*t*	95% CI	*β*	*t*	95% CI
Gender	0.336	8.217 ***	[0.256, 0 0.416]	0.165	4.287 ***	[0.089, 0.240]	0.235	7.283 ***	[0.171, 0.298]	0.104	3.302 **	[0.042, 0.166]
Age	−0.035	−2.339	[−0.064–0.006]	−0.024	−1.687	[−0.051, 0.004]	−0.016	−1.373	[−0.039, 0.007]	−0.015	−1.383	[−0.037, 0.006]
Autonomous fitness behavior	0.416	23.935***	[0.382 0.450]	0.301	18.487***	[0.269, 0.333]	0.198	12.583***	[0.167, 0.229]	0.171	10.564***	[0.140, 0.203]
Self-control	——	——	——	—	——	——	0.281	10.515***	[0.228, 0.333]	0.080	2.978 ***	[0.027, 0.133]
Exercise identity	——	——	——	—	——	——	——	——	——	0.779	25.435***	[0.719, 0.840]
*R*^2^	0.630			0.576			0.624			0.817		
*F*	212.564***			154.528***			178.67***			387.369***		

**p < 0.01, ***p < 0.001, 95%CI: 95% Confidence Interval.

Model 1 indicated that autonomous fitness behavior could directly predict mental health literacy (*β* = 0.416, 95%CI [0.382,0.450], *p* < 0.001). Model 2 showed that autonomous fitness behavior positively predicted self-control (*β* = 0.301, 95%CI [0.269, 0.333], *p* < 0.001). The results of Model 3 demonstrated that autonomous fitness behavior positively predicted exercise identity (*β* = 0.198, 95%CI [0.167,0.229], *p* < 0.001), and self-control could directly and positively predict exercise identity (*β* = 0.281, *p* < 0.001). Model 4 revealed that autonomous fitness behavior, self-control, and exercise identity simultaneously exhibited positive predictive effects on mental health literacy (*β* = 0.171, 95%CI [0.140,0.203], *p* < 0.001; *β* = 0.080, 95%CI [0.027, 0.133], *p* < 0.001; *β* = 0.779,95%CI [0.719,0.840], *p* < 0.001).

The results showed no significant differences in key variables across age groups, while gender differences were significant-males scored higher than females in autonomous fitness behavior, self-control, and exercise identity.

**Figure 2:**
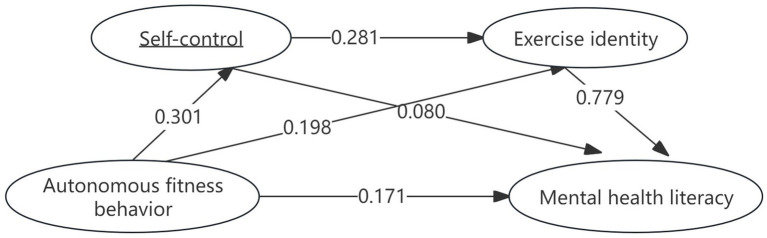
The chain-mediated mediation path of autonomous fitness behavior to mental health literacy.

The results of the further chain mediation model test are presented in Table 4 and Figure 2. Specifically, the indirect effect of the path with self-control as the mediating variable was 0.024 (95% CI = [0.008, 0.042]), the indirect effect of the path with exercise identity as the mediating variable was 0.155 (95% CI = [0.122, 0.189]), and the indirect effect of the path with both self-control and exercise identity as mediating variables was 0.066 (95% CI = [0.049, 0.084]). The cumulative total of all indirect effects was 0.245 (95% CI = [0.210, 0.279]), with the three indirect paths accounting for 5.77, 37.25, and 15.87% of the total effect, respectively. These findings collectively confirm the establishment of a chain mediating effect in the relationship between perceived social support and autonomous fitness behavior.

**Table 4:** Bootstrap analysis of significance test of intermediary effect.

Influence path	Indirect effect	95% confidence interval			Ratio of total effect
		BootSE	BootLLCI	BootULCI	
Total indirect effect	0.245	0.017	0.210	0.279	58.89
Ind1	0.024	0.009	0.008	0.042	5.77
Ind2	0.155	0.017	0.122	0.189	37.25
Ind3	0.066	0.009	0.049	0.084	15.87
Ind1–Ind2	−0.131	0.021	−0.171	−0.091	–
Ind1–Ind3	−0.042	0.011	−0.065	−0.019	–
Ind2–Ind3	0.089	0.022	0.046	0.132	–

Ind1: autonomous fitness behavior → self-control → mental health literacy; Ind2: autonomous fitness behavior → exercise identity → mental health literacy; Ind3: autonomous fitness behavior → self-control → exercise identity → mental health literacy. BootLLCI = Lower limit, BootULCI=Upper limit, BootSE = Standard Error.

## Discussion

### The relationship between autonomous fitness behavior and mental health literacy

The findings of this study demonstrate a significant positive association between autonomous fitness behavior and mental health literacy among college students, corroborating existing theoretical frameworks and empirical evidence. This section expands on the mechanisms and contextual factors underlying this relationship, integrating multidisciplinary perspectives to provide a comprehensive explanation.

First, Autonomous fitness behavior, defined by self-determined engagement in physical activity, elicits neurobiological responses that directly enhance mental health literacy. Regular exercise stimulates the release of endorphins, serotonin, and brain-derived neurotrophic factor (BDNF), which collectively improve mood, reduce stress, and support cognitive function (Mandolesi et al., 2018). Unlike externally mandated exercise, autonomous participation amplifies these effects by minimizing perceived exertion and increasing enjoyment (Teixeira et al., 2012). This neurochemical modulation enhances emotional regulation-a critical dimension of mental health literacy-enabling students to better manage academic and social stressors (Schuch et al., 2016).

Second, Self-Determination Theory (SDT) offers a robust framework for interpreting these findings. Autonomous fitness behavior fulfills three fundamental psychological needs: autonomy (volitional choice), competence (perceived efficacy), and relatedness (social connection) (Deci and Ryan, 2008). When students engage in fitness activities out of intrinsic motivation, they develop a stronger sense of self-efficacy and mastery, which are pivotal for mental health literacy (Wang et al., 2025). For example, prior research indicates that autonomously motivated individuals exhibit greater resilience and adaptive coping strategies, as their engagement aligns with personal values rather than external pressures (Ng et al., 2012).

Furthermore, Autonomous fitness behavior fosters long-term adherence to exercise, which in turn cultivates psychological flexibility and self-regulation-key components of mental health literacy. Consistent participation in self-directed physical activity reinforces goal-setting, perseverance, and adaptive problem-solving skills (Baumeister et al., 2010). These competencies extend beyond fitness, equipping students with strategies to navigate academic pressures and interpersonal challenges (Kashdan and Rottenberg, 2010). Longitudinal studies suggest that such behavioral consistency predicts sustained improvements in mental health awareness and self-management (Chekroud et al., 2018).

Finally, the social context of autonomous fitness further amplifies its mental health benefits. Group-based or community-oriented fitness activities provide social support, reinforcing both exercise adherence and psychological well-being (Haslam et al., 2016). Additionally, voluntary engagement fosters the development of an “exerciser identity,” wherein students internalize fitness as part of their self-concept (Strachan et al., 2009). This identity integration enhances mental health literacy by promoting proactive health behaviors and increasing awareness of psychological well-being strategies.

The relationship between autonomous fitness behavior and mental health literacy is mediated by neurobiological, psychological, behavioral, and social mechanisms. These pathways collectively illustrate how self-determined physical activity enhances students’ capacity to understand, regulate, and improve their mental health. Future interventions should leverage these insights by promoting autonomy-supportive fitness environments and integrating mental health education into physical activity programs.

### The mediating role of self-control

The present findings establish self-control as a significant mediator in the relationship between autonomous fitness behavior and mental health literacy, with an indirect effect of 0.024 accounting for 5.77% of the total effect. This dual-pathway influence suggests that while autonomous fitness directly enhances mental health literacy, it also confers indirect benefits through strengthening self-regulatory capacity.

The mediating mechanism operates through two synergistic psychological processes. First, the very nature of autonomous fitness cultivates self-control through repeated self-regulation. When students voluntarily engage in physical activity, they must consistently overcome immediate gratifications (e.g., sedentary behaviors) and environmental barriers to maintain their exercise regimen. This ongoing process of goal-directed self-regulation serves as an implicit training ground for self-control. As Tangney et al. (2004) demonstrated, such regular practice of delaying gratification and persisting toward long-term objectives effectively strengthens executive function and impulse control. The intrinsically motivated nature of autonomous fitness may be particularly conducive to this developmental process, as it fosters greater engagement in self-regulatory efforts compared to externally imposed exercise routines.

Second, enhanced self-control facilitates mental health literacy by improving emotional and behavioral regulation. Individuals with stronger self-control exhibit superior affect modulation, demonstrating reduced emotional reactivity to stressors and more adaptive coping strategies (Hofmann et al., 2012). These regulatory skills directly translate to key dimensions of mental health literacy, including: Improved recognition of emotional states, Enhanced capacity for cognitive reappraisal of stressors, Greater behavioral flexibility in challenging situations, For instance, when facing academic pressures, students with developed self-control can better implement evidence-based stress management techniques rather than resorting to maladaptive avoidance behaviors (Oaten and Cheng, 2006). This transfer of regulatory competencies from the fitness domain to broader psychological functioning represents a crucial pathway through which autonomous fitness promotes mental health awareness and skills.

The current study identifies self-control as an independent mediator between autonomous fitness behavior and mental health literacy, yet its mediating effect (5.77%) is far lower than that of exercise identity (37.25%). This relatively modest mediation proportion suggests self-control operates primarily as a foundational capacity that enables other mechanisms (e.g., exercise identity formation) rather than serving as the predominant mediator—aligning with contemporary models viewing self-control as a necessary but insufficient factor for sustained behavior change (Inzlicht et al., 2021). Two context-specific factors for Chinese college students further explain this weak mediating role: First, their relatively high baseline self-control-developed through preparing for the competitive Gaokao and honed in secondary education-limits regulatory space, as autonomous fitness and mental health literacy regulation both rely on “above-baseline” resources, reducing self-control’s marginal contribution. Second, heavy academic pressure consumes self-control resources; per the “self-control resource model,” when most resources go to academics, the remaining capacity to mediate the link between autonomous fitness and mental health literacy shrinks-unlike exercise identity, which is less dependent on concurrent resource allocation. Overall, the finding underscores the importance of considering self-control within a network of psychological processes rather than in isolation when examining its role in mental health outcomes.

### The mediating role of exercise identity

The current findings reveal exercise identity as a robust mediator in the autonomous fitness-mental health literacy relationship, accounting for 37.25% of the total effect - substantially more influential than self-control’s mediating role. This substantial mediation occurs through two synergistic psychological processes that warrant detailed examination.

First, autonomous fitness behavior systematically cultivates exercise identity through repeated self-concept reinforcement. When individuals engage in physical activity driven by intrinsic motivations (e.g., enjoyment, personal growth), they undergo a gradual process of identity crystallization (Strachan et al., 2025). Each autonomous exercise session functions as an identity-relevant experience that validates one’s self-perception as an exerciser, strengthens emotional connections to physical activity, and reinforces exercise-related values and beliefs. This process aligns with identity theory’s proposition that repeated role-consistent behaviors solidify corresponding identities (Stets and Burke, 2014a,b). The voluntary nature of autonomous fitness is particularly potent for identity formation, as it reflects authentic personal choice rather than external imposition.

Second, an established exercise identity enhances mental health literacy through multiple pathways: self-efficacy development, whereby regular exercise accomplishments provide mastery experiences that generalize to other life domains (Bandura, 1997), equipping individuals with confidence to manage psychological challenges; emotional regulation, through which the neurobiological benefits of consistent physical activity (e.g., endorphin release, HPA axis modulation) become associated with one’s identity, creating sustainable mood regulation patterns (Dishman et al., 2006); and social reinforcement, as exercise identity often develops within social contexts (e.g., sports teams, fitness communities), providing both instrumental support and normative expectations that promote mental health behaviors (Cruwys et al., 2014). It also boosts well-being self-efficacy, as fitness achievements tied to exercise identity build confidence in overcoming difficulties, which translates to trusting one’s ability to manage emotions and reinforces practical mental health literacy (Cui and Zhou, 2025).

The particularly strong mediating effect (37.25%) suggests exercise identity operates as a central hub that transforms transient fitness behaviors into enduring psychological resources. Unlike self-control’s regulatory function, exercise identity works through deeper self-concept reorganization, making its effects more comprehensive and sustainable (Wang et al., 2024). This explains why it accounts for nearly seven times more variance than self-control in the mediation model.

### The chain mediating effect of autonomous fitness behavior and mental health literacy

The current study demonstrates a significant sequential mediation pathway in which autonomous fitness behavior enhances mental health literacy through the chain of self-control and exercise identity, accounting for 15.87% of the total effect (indirect effect = 0.066). This finding reveals a dynamic psychological transformation process that progresses from behavioral regulation to identity formation, ultimately contributing to mental health competence.

The initial phase involves autonomous fitness serving as a training ground for self-control development. The voluntary nature of this activity requires individuals to consistently regulate their time, energy, and impulses - such as resisting the temptation to skip workouts for immediate gratification. Neuroscientific evidence suggests this repeated self-regulation strengthens prefrontal cortical networks and frontostriatal connectivity, which are critical for executive function (Berkman et al., 2012; Stillman et al., 2020). This enhanced self-control capacity then creates the necessary conditions for sustained exercise adherence, a prerequisite for identity formation.

As individuals maintain consistent fitness routines through improved self-control, they enter the identity crystallization phase. Each successful workout serves as behavioral verification that reinforces their self-concept as an exerciser (Crone and Drunen, 2024). Progressive skill mastery and social recognition within fitness communities further consolidate this identity. The transition from behavioral consistency to stable identity represents a crucial psychological shift where exercise moves from being an activity one does to becoming part of who one is.

The final phase sees the established exercise identity operating as psychological capital that enhances mental health literacy (Shrestha et al., 2025). This occurs through multiple pathways: the cognitive benefits of overcoming physical challenges build resilience; the physiological effects of regular exercise improve stress modulation; and the social aspects of fitness communities provide emotional support. The 15.87% mediation weight underscores the importance of this transformation sequence, revealing how momentary acts of self-regulation can, through identity formation, generate broad psychological benefits that extend far beyond the gym.

The significant gender differences (males outperforming females in autonomous fitness behavior, self-control, and exercise identity) may be attributed to two contextual factors: first, traditional gender norms in China often associate physical activity with masculinity, leading males to have stronger intrinsic motivation for fitness and more positive self-perception as “exercisers”; second, college females are more likely to face social or self-imposed constraints (e.g., concerns about body image, lower willingness to participate in group fitness), which reduce their autonomous fitness engagement and further weaken the development of exercise identity and self-control in the fitness context. These factors collectively contribute to the observed gender disparities in key variables.

## Limitations and future prospects

The strengths of this study are as follows: First, it focuses on college students as a specific group, filling the research gap in the subfield of the association between autonomous physical activity and mental health among this population. Second, the research topic closely aligns with the needs of health promotion for adolescents in the field of public health, thereby possessing clear practical relevance and value, yet several limitations require consideration.

First, the cross-sectional design prevents causal inferences. While the proposed mediation model is theoretically sound, longitudinal or experimental studies are needed to confirm temporal relationships and directional effects among autonomous fitness behavior, self-control, exercise identity, and mental health literacy. For example, we cannot definitively conclude that autonomous fitness behavior precedes and drives improvements in self-control, nor can we exclude reverse causality (e.g., individuals with higher mental health literacy may be more likely to initiate and sustain autonomous fitness behavior). Future research could use multi-wave longitudinal designs to track changes in these variables or randomized controlled trials (RCTs) to test whether autonomy-supportive fitness interventions enhance mental health literacy, and compare findings with previous studies.

Second, reliance on self-report measures may introduce common method bias, despite Harman’s single-factor test ruling out significant issues. Self-reported data are prone to social desirability bias and recall errors, particularly for constructs like self-control and exercise identity. Thus, future studies should integrate objective assessments (e.g., wearable devices for fitness behavior tracking, laboratory-based tasks for self-control) alongside self-reports; peer or instructor ratings of exercise identity could also improve assessment comprehensiveness.

Third, the sample, restricted to college students in Shandong Province, China, limits generalizability. Shandong Province was selected primarily due to its representative regional characteristics (balanced distribution of higher education institutions and distinct cultural traits), but regional differences in fitness culture and educational pressures mean findings may not generalize to other provinces. To address this, future research should expand sample size and increase diversity: on one hand, extend sampling to multiple provinces to capture regional variations; on the other hand, achieve a balanced gender ratio via stratified sampling (based on college students’ gender distribution in each region) to avoid gender imbalance biases, ensuring broader applicability to Chinese college students.

Fourth, unmeasured confounding variables (e.g., baseline mental health, socioeconomic status, access to fitness facilities) could affect the studied relationships-for instance, students with higher socioeconomic status may have better access to fitness resources and higher mental health literacy. Future studies should include these covariates to refine the model and identify responsive subgroups. Finally, self-control and exercise identity only partially explained the total effect, indicating unexamined mechanisms (e.g., social support, psychological need satisfaction). Expanding the model to include these variables would provide a more holistic understanding of pathways.

Building on these limitations, additional research avenues emerge. First, develop experimental interventions (e.g., gamified fitness apps, choice-based exercise programs) to foster autonomous fitness behavior, and evaluate their impact on key variables. Second, use qualitative approaches (e.g., interviews, focus groups) to explore students’ subjective experiences, complementing quantitative insights. Third, conduct interdisciplinary research (integrating psychological, physiological, and social perspectives) to reveal interactions between biological markers and psychological mediators. Additionally, explore moderators (e.g., gender, conscientiousness) to identify boundary conditions, and integrate technology (e.g., wearables, ecological momentary assessment) to capture real-time dynamics between fitness behavior and mental health indicators.

## Conclusion

College students’ autonomous fitness behaviors can not only independently predict mental health literacy, but also indirectly predict mental health literacy through the mediating effect of self-control and exercise identity, as well as the chain intermediary role of self-control and exercise identity. These findings not only enrich the relevant theoretical frameworks in the field of autonomous fitness behavior and mental health literacy but also provide empirical support for designing targeted interventions to enhance college students’ mental health literacy.

## Data Availability

The datasets presented in this study can be found in online repositories. The names of the repository/repositories and accession number(s) can be found in the article/Supplementary material.
